# On the Reuse of SLS Polyamide 12 Powder

**DOI:** 10.3390/ma15165486

**Published:** 2022-08-10

**Authors:** Pedro C. Gomes, Oscar G. Piñeiro, Alexandra C. Alves, Olga S. Carneiro

**Affiliations:** 1 IPC/LASI—Institute for Polymers and Composites, University of Minho, 4800-058 Guimarães, Portugal; 2 CMEMS—Center for MicroElectroMechanical Systems, University of Minho, 4800-058 Guimarães, Portugal; 3 LABBELS—Associate Laboratory, 4800-058 Guimarães, Portugal; 4 NM3D Ibérica—Sistemas de Metrologia Industrial, 4920-071 Vila Nova de Cerveira, Portugal; 5 IBTN/Euro—European Branch of the Institute of Biomaterials, Tribocorrosion and Nanomedicine, Department of Mechanical Engineering, University of Minho, 4800-058 Guimarães, Portugal

**Keywords:** Selective Laser Sintering, Polyamide 12, powder reuse, morphology, mechanical properties, milling and filtering

## Abstract

In the Selective Laser Sintering (SLS) technique, the great majority of the powder involved is not included in the final printed parts, being just used as a support material. However, the quality of this powder is negatively affected during the process since it is subjected to high temperatures (close to its melting temperature) during a long time, i.e., the printing cycle time, especially in the neighborhood of the printed part contour. This type of powder is relatively expensive and large amounts of used powder result after each printing cycle. The present paper focuses on the reuse of Polyamide 12 (PA 12) powder. For this sake, the same PA 12 powder was used in consecutive printing cycles. After each cycle, the remaining non-used powder was milled and filtered before subsequent use. Properties of the powder and corresponding prints were characterized in each cycle, using differential scanning calorimetry (DSC), scanning electron microscopy (SEM), computed tomography (CT), and tensile tests. It was concluded that subjecting the same powder to multiple SLS printing cycles affects the properties of the printed parts essentially regarding their morphology (voids content), mechanical properties reproducibility, and aesthetical aspect. However, post-processing treatment of the powder enabled to maintain the mechanical performance of the prints during the first six printing cycles without the need to add virgin powder.

## 1. Introduction

Additive Manufacturing (AM) technologies have been gaining prominence in the industrial world and are used to create functional parts, reaching geometries impossible to achieve by conventional manufacturing technologies [1,2]. More commonly known as 3D printing or Rapid Prototyping (RP), the term additive manufacturing refers to any technology based on the production of parts layer-by-layer from a computer-aided design (CAD) 3D model previously created [3,4,5]. Dating back to the 80 s, the first commercially available AM technology consisted of the use of a UV source to harden a UV-sensitive polymer (photopolymer) to build the desired structure, this being the first Stereolithography (SLA) equipment in this field. Even though it was innovative, this technology was also very expensive and not reliable [6,7,8]. One variant belonging to this particular family of technologies is the Selective Laser Sintering (SLS) process. SLS is becoming popular and is no more limited to high-tech industry. It is being increasingly chosen to manufacture functional parts, with great dimensional precision, using a wide variety of materials for different application areas (like automotive, aerospace, and medical among many others) and a relatively low cycle time [3,9].

Beyond the geometrical freedom, the use of SLS, and AM techniques in general, brings other advantages, such as no need for molding tools, and removing constraints normally associated with the conventional subtractive processes [10].

SLS is a technique based on the sintering of selected zones of a build surface of the chosen material, through a laser beam, usually in an oxygen-free build space.

The solid polymeric part is made layer-by-layer, where a feed cartridge supplies the required amount of powder and a layering roller spreads the powder, covering the build platform, which descends when the feed cartridge ascends. Through a conversion software, an STL file is transformed into code lines that will guide a laser (commonly a CO_2_ one) to focus on the desired powder grains, breaking their surface tension and sintering them together, creating a sintered solid part. Since the build space is heated to a temperate just below the melting temperature of the powder material, the energy supplied by the laser is just the additional one required to melt the powder particles. Maintaining the build space heated and subsequently slowly cooling it also helps to minimize residual stresses of thermal origin in the printed parts, preventing warping and distortion. The accuracy of this process is mainly dependent on the raw material used, motivating the development of new powders [1,11,12,13]. However, there are some limitations regarding to the type of polymers available for this technology, the market being dominated by polyamides. When compared with Fused Filament Fabrication, FFF, the printing speed, the easiness of process control, the higher dimensional accuracy, and the capability to self-sustain the structures it builds make the SLS stand out as an excellent productive option, reducing the post-processing tasks and increasing productivity [14,15].

One of the most critical limitations of this technique is the fact that 80–90% of the powder material present in the build chamber is not sintered and may be deteriorated, jeopardizing its reuse. For example, agglomeration of the powder reduces its flowability, making it hard to build a homogeneous build surface. Moreover, a decrease in the physical and mechanical properties of the powder can occur, compromising the quality of the printed parts [12,16]. Therefore, there is a great potential for powder waste that has negative economic and environmental impacts. A common strategy employed to minimize these impacts is the reuse of this powder. However, the high temperatures at which the powder is maintained in the build space (close to its melting temperature, i.e., around 180 °C in the case of PA 12) and the long residence time (for example, a minimum of 3 h in the equipment used in this work) may deteriorate the properties of the powder, limiting the maximum number of printing/reusing cycles and/or incorporation rates [17].

PA 12 is the most common polymer used in the SLS technique, mainly due to its easy and flexible processing. This is a consequence of the large temperature range it presents between the start of melting during the heating process and the start of crystallization in the cooling process. This allows keeping the material heated without crystallization until the cooling stage, maximizing consolidation and preventing warpage [16,18]. Other properties that make this material popular are its better mechanical properties and low moisture absorption, when compared with other polyamides, good resistance to abrasion, and good resistance to chemicals including solvents [18].

PA 12 is a semi-crystalline condensation polymer having amide bonds in the main chain. Some of its functional groups may contain open-end chains, which may be susceptible to post-condensation and crosslinking reactions when subjected to high temperatures.

In the SLS process, there are two main types of potential powder waste: that remaining unsintered in the build space and the excess powder used to build each layer (that returns to the feed cartridges), called feed material. The powder in build space is the one that suffers the most severe thermal effects and, consequently, aging/degradation, once it remains at high temperatures longer than the feed powder. The aging of the powder depends mainly on the build chamber temperature and residence time. Previous studies showed that the predominant degradation processes occurring in polyamides are the cross-linking and chain scission [8].

One way to reduce the negative effects of powder degradation is using flow and antistatic agents, intended to increase its flowability. These additives have a very small particle size (around 1 μm) and are incorporated at very low rates, between 0.05 and 1.5 wt. %. Their role is to eliminate the static charge present in the powder, reducing its tendency to agglomerate and improving its flowability. Nevertheless, these agents may negatively affect the mechanical integrity of printed parts, a disadvantage that must be considered [14].

On the other hand, low-temperature SLS can also minimize the powder degradation. As the name suggests, this technique is based on the maintenance of the powder at lower temperatures, reducing the degradation of the material. Although this might sound simple, it increases the chance of curling, especially if the printed parts have thin walls. Binding the parts to a rigid base might reduce this tendency, but it will demand more post-printed processes to finish the parts [16].

Finally, the most common method used to reduce the loss of material properties is to add virgin powders to the reused ones. Normally, refresh rates of 50% are used in this technique. Studies differ in this subject, since some authors argue that the powder that remains in the build space should not be reused, and others state that this material can be reused, but regular check-up tests must be carried out to assess its quality. A common test used to assess the integrity of the material is the Melt Flow Rate (MFR)/Melt Flow Index (MFI), which in the case of PA 12 must be maintained above 18 g/10 min [12,16].

The degradation, or aging, of polymers can be promoted by internal or external causes [19,20]. The external causes are considered to be physical and chemical interactions of the material with its surroundings, as for example, weathering, UV radiation, humidity, and temperature, the latter being the most notable degrading agent of this type. The internal causes are thermodynamically unstable states present in the material that, if activated, usually by thermal stress, lead to a measurable change in properties. Examples of internal causes are unstable crystallization states, residual stresses, and incomplete polycondensation [19,20,21].

In the case of PA 12, the main degradation mechanism is the cross-linking of polymer chains caused by oxidation, which is said to be dominant at the initial stages of the degradation process. The main consequences of the degradation are a decrease in flowability of the powder, due to an increase in molecular weight, and poor mechanical properties, especially strain at break and maximum stress [12,22]. The increase of molecular weight can also occur by post condensation reactions. This leads to a shift of the crystallization temperature to lower values, due to the lower chain mobility. Although this broadens the processing window, higher molecular weight results in higher viscosity, which prejudices the spreading of the powder and makes the SLS printing process harder [23]. Chain scission may also occur, but at later stages of the degradation process, causing a decrease in molecular weight, counterbalancing the increase of molecular weight caused by cross-linking [23]. Studies showed that the increase of molecular weight affects the quality of manufactured parts, both in the correct set up of the build surface and in the reproducibility of the printed parts [23].

In the literature, there is a proposed methodology that aims to predict the property loss of polymers when subject to reprocessing through primary recycling, in processes such as injection molding. In this case, the virgin polymer is processed (i.e., a part is injected), reground, mixed with virgin polymer, and reprocessed. This methodology, developed by Bernardo et al. [24], was able to predict the material properties decay and relies on two simplifying assumptions: (i) the regrind process does not affect the properties of the polymer (it is only intended to enable feeding the material to the injection molding machine), and (ii) the fraction of the virgin polymer added in each cycle is constant. Very recently, Lopes et al. [25] successfully applied this methodology to SLS PA 12 printed parts, predicting the decay of the Young’s modulus, tensile stress at yield, and tensile stress at break. The authors used 5 cycles of reprocessing and performed a fitting for the linear law of mixtures, through a custom-made software. This software adjusted 3 different models and predicted the decay of properties for the first 20 reprocessing cycles. The main conclusions of this work were that the mechanical properties and the density of the printed samples were compromised when only reprocessed material was used, even at the earlier stages of reprocessing and that the loss of properties can be minimized if a virgin powder fraction is incorporated in the mixture.

The aging of PA 12 powder exposed to multiple processing cycles and subjected to a post-processing treatment after each printing cycle, namely, filtering, milling, and homogenization, is the aim of the current study. Therefore, the use of the methodology employed in [25] is not valid since after each printing cycle the lower quality unsintered powder is improved and/or rejected.

Another factor that may influence the behavior of the printed parts is the orientation in which they are printed. Tomanik et al. [26] studied the differences in mechanical behavior of PA 12 printed samples with different building orientations relative to the build platform. They concluded that the samples printed at 0° (i.e., horizontally, parallel to the build platform) present the most ductile behavior, and those printed at 45° showed the most brittle one. However, none of the printing orientations originated a Young’s modulus close to that indicated by the supplier. This will also be investigated in the present work.

This work was carried out in the frame of a partnership between a university and an industrial company that provides SLS printing services.

## 2. Materials and Methods

### 2.1. Polyamide 12 Powder

PA12-L 1600 powder (from Prodways Technologies, Paris, France), whose main properties are listed in Table 1, was used to produce all the samples.

### 2.2. Processing Equipment and Methodology

**Figure 1:**
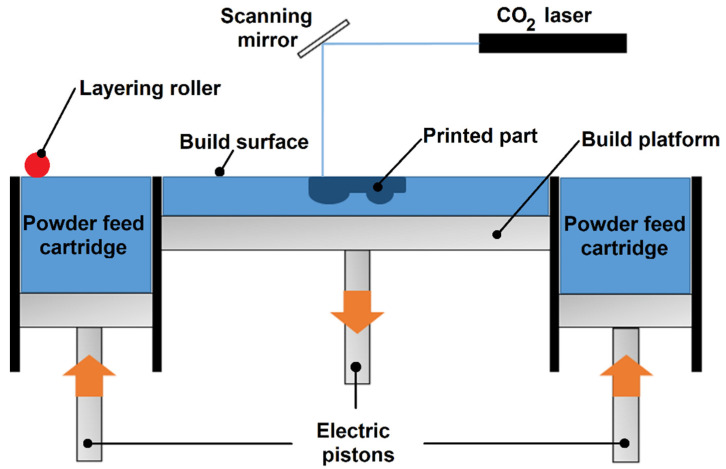
Schematic view of the SLS equipment used.

The SLS equipment used for the production of samples was a ProMaker P1000 (Prodways Technologies), schematically shown in Figure 1, and the main specifications are listed in Table 2.

The parameters used in all the printing cycles were fixed and chosen based on previous knowledge of the company. The heating and cooling stages had a 90 min duration, while the printing cycle was fixed at around 9 h, including warm-up and cooling stages. The SLS processing parameters are given in Table 3.

The unsintered powder milling (through sieves and ceramic balls) and filtering were performed in the ProTool BS01 equipment (Prodways Technology), employed to destroy agglomerates and to separate the bigger remaining ones and/or pre-sintered material (the last filter has a 200 µm mesh), for later disposal. The resulting powder was homogenized in an intensive mixer, having three rotational blades, for 2 min.

Lastly, printed parts were cleaned by sand jet in a Guyson Formula 1400 equipment (Prodways Technologies).

**Figure 2:**
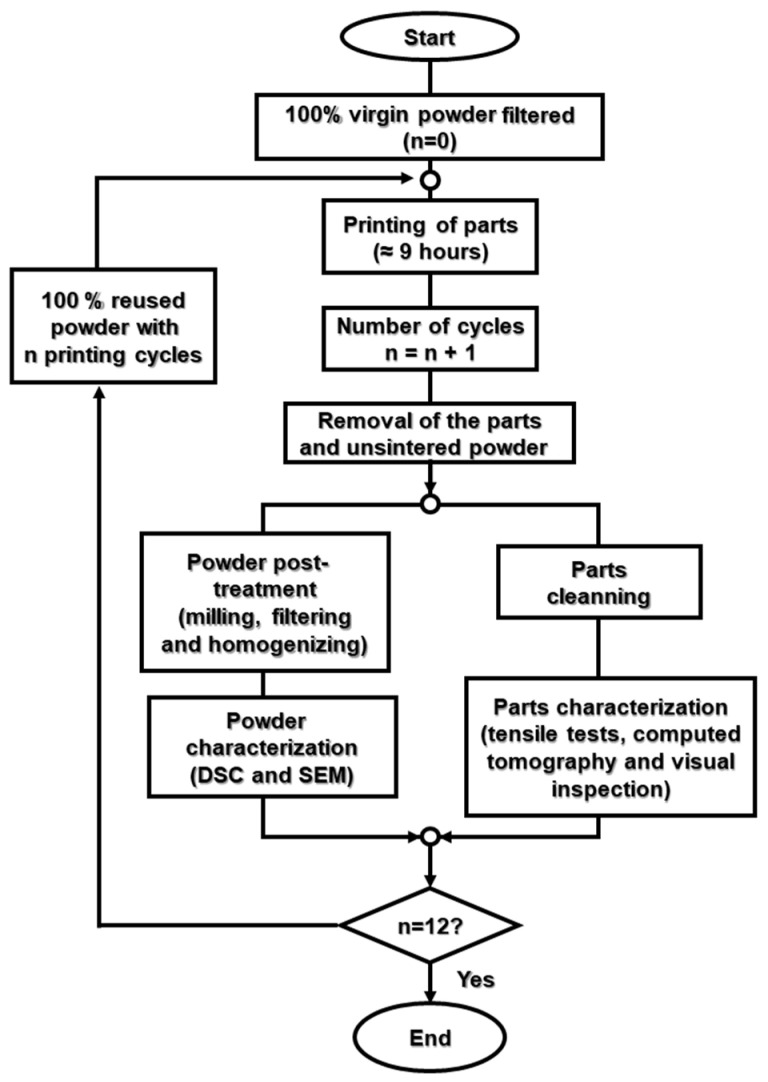
Sequence and main stages involved in the methodology used. Note: ‘n’ is the number of processing/printing cycles and is used to name powder and printed parts.

The methodology followed in this work is illustrated in Figure 2, where the sequence of the main stages is depicted.

In each printing cycle, other parts (with commercial interest for the industrial partner) were added to the build space, in order to reach the fixed cycle duration of around 9 h. It is important to stress that no virgin material was added after each cycle. Therefore, to ensure that there was enough amount of powder for all the cycles performed (twelve, in total), the equipment was loaded to its maximum capacity on the first processing cycle, even though there was only a small portion of powder used for printing. Furthermore, the test samples were always printed in the same coordinates of the building space.

### 2.3. Characterization of the Powder and Printed Parts

Differential Scanning Calorimetry analyses (DSC) were performed in a Netzsch DSC 200 F3, to determine the melting temperature and the melting enthalpy of the powder along subsequent processing cycles. The samples heating was carried out from 20 °C to 220 °C, at a rate of 10 °C/min and maintained at 220 °C during 1 min. Then, the samples were cooled down until 20 °C at a rate of 10 °C/min. Nitrogen, purged at 50 mL/min, was used to ensure an inert environment.

Scanning Electron Microscopy (SEM) using FEG-SEM FEI Nova 200 field emission gun scanning electron microscope, from FEI Company, Hillsboro, OR, USA, was used to characterize the powder morphology. High voltage of 10 kV and secondary electrons (SE) mode were selected for the analysis.

**Figure 3:**
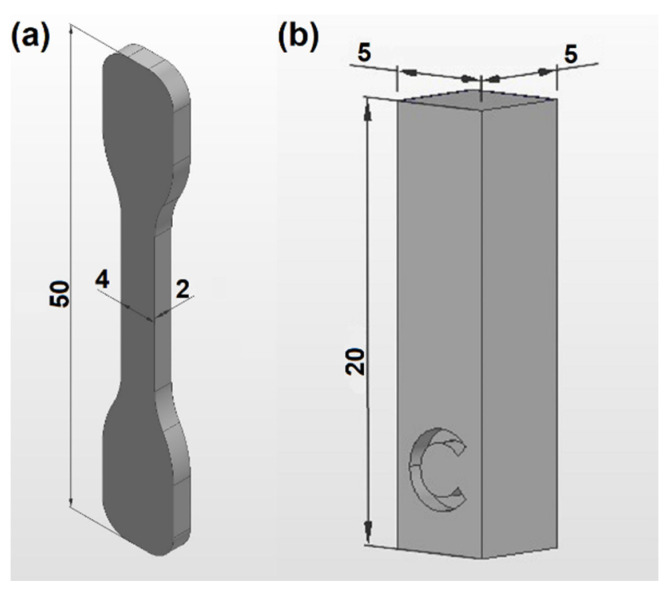
Parts printed in each (re)processing cycle: (**a**) tensile test sample and (**b**) CT sample for porosity characterization (dimensions in mm).

For characterization purposes, two distinct sample geometries were produced for each printing cycle:

(i) Samples for tensile tests, shown in Figure 3a, according to standard DIN 53504-S3a, were printed in two different orientations (vertical, V, and horizontal, H, relative to the build platform). These samples (7 in each orientation) were printed inside two different cages (printed in the same cycle), marked with V and H to distinguish the vertical and horizontal orientations. Marking the cages instead of the samples intended to avoid any eventual negative effect on the samples’ mechanical performance.
(ii) Samples for porosity analysis to be performed with computed tomography (CT), printed vertically in the center of the build platform and marked with a C symbol, as illustrated in Figure 3b.

The coordinates of the samples in the build space were kept fixed in all the printing cycles (corresponding to the first layers of the center of the build space).

A Shimadzu AG-X tester was used to conduct the tensile tests until break. The deformation was monitored through a video extensometer. The tests were performed at a 10 mm/min speed, at room temperature, and a load cell of 1 kN was used.

**Figure 4:**
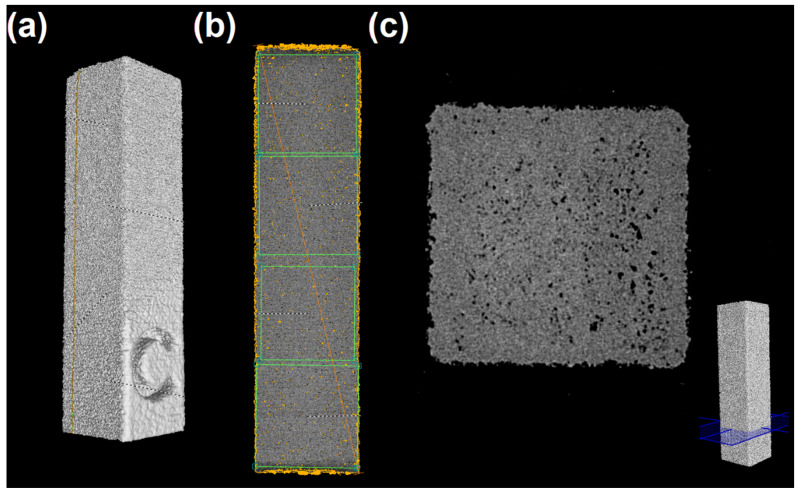
Computed tomography analysis: (**a**) 3D view; (**b**) one of the three longitudinal cuts made in each sample, showing the four sections where porosity values were taken; and (**c**) one of the three transversal cuts made for sample shape checking.

The printed samples illustrated in Figure 3b were analyzed in a computed tomography equipment, XT H 225 S, from Nikon Metrology, using a tungsten filament. The 360° scans were performed with beam energy of 180 kV, power beam current of 11 µA, 20 W of power, and exposure of 4 fps. Two frames were taken for each projection. All images were analyzed in the Visual Graphics Studio software. For each sample, three longitudinal cuts (see Figure 4a) performed at 1.0, 2.5, and 4.0 mm from the sample wall marked with a C symbol, were made, and four areas were considered in each section (see Figure 4b). Thus, a total of twelve values of porosity were determined for each sample. Additionally, three transversal cuts (see Figure 4c) were made to check the shape of the samples. The porosity results are presented as the average values from three independent samples. Statistical analysis was evaluated by one-way ANOVA followed by Tukey’s test for multiple comparisons, considering *p* < 0.05 as significant.

During the successive printing cycles, a visual inspection of the parts was carried out to qualitatively evaluate their aesthetic quality. This is a relevant issue since SLS is often used to produce parts for applications where good surface finishing is required.

## 3. Results and Discussion

### 3.1. Powder

**Figure 5:**
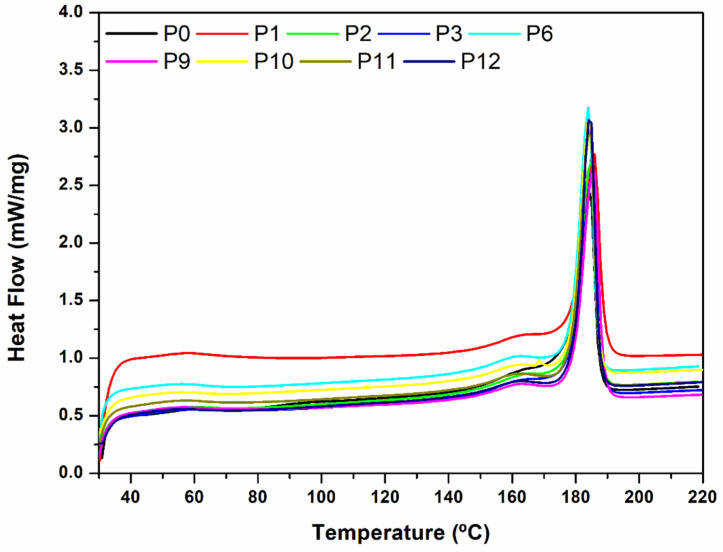
DSC heat flow curves obtained for the virgin powder (0 cycles, P0 in the graph) and for reused powder collected after some of the 1 to 12 cycles (samples P1 to P12 in the graph).

**Figure 6:**
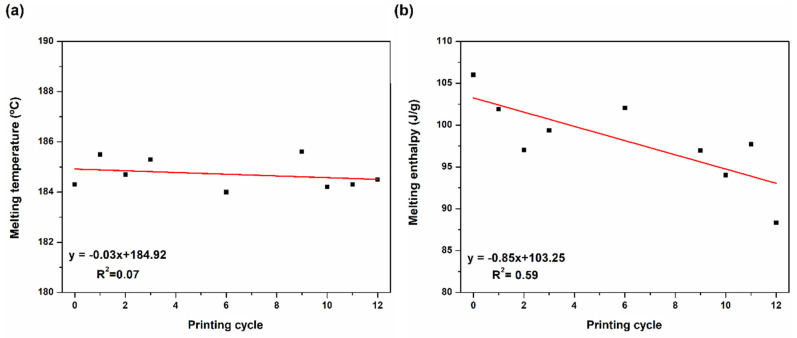
Powder DSC results: (**a**) melting temperature and (**b**) melting enthalpy.

DSC characterization was only performed on the powder, the raw material of the printing stage, since it is the eventual degradation of this system that will influence the printed parts characteristics. As can be seen in Figure 5, the DSC curves of the powder tested along the printing cycles maintain a similar trend. This means that there are no significant changes occurring in the material. This was also concluded in a recent work [25], but the detailed results were not shown. In the current work, the evolution of the melting temperature and melting enthalpy is shown in Figure 6a,b, respectively. The melting temperature is almost maintained (there is a 1.4 °C difference between the limiting values measured), showing an overall slight tendency to decrease along the twelve successive printing cycles. However, in some intermediate cycles the value of this temperature is higher than that corresponding to the virgin powder (named as 0). This is in line with the recommendation made by most powder suppliers to maintain, or even slightly increase, the temperature of the SLS process when reused powder is employed. The melting enthalpy shows the same trend, but more markedly, presenting a variation from 106 J/g to 88 J/g (corresponding to a variation in the degree of crystallization from around 43% to 36%). These results are in line with those from [25] and suggest that chain-scission degradation mechanism may be the prevalent one in the first printing cycles. Heat promotes intra-chain bonds stresses, due to higher amplitude intramolecular vibrations, which may cause their rupture (chain-scission) originating fragments and radical end-groups [27,28]. The scatter of the results obtained is also worth noticing. This may be originated by the fact that the powder is still not homogeneous, despite the post-processing treatment it is subjected to. In fact, the powder particles experience different thermal histories during the printing stage, depending on their distance to the laser beam (printed parts contour).

**Figure 7:**
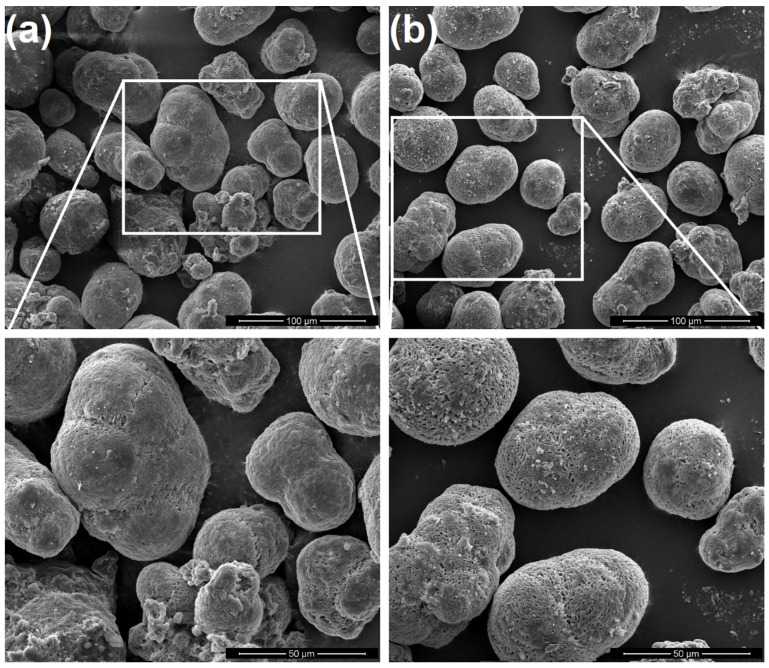
SE/SEM images for: (**a**) virgin powder and (**b**) powder after 12 printing cycles.

Figure 7 shows SE/SEM images of the virgin and after printing cycle 12 powders. It can be observed that the reused powder has a rougher surface and seems to have a higher porosity, which might also induce some porosity in the produced parts. Dadbakhsh et al. [18] also observed an increment in porosity in aged PA 12 powder. These authors attributed this behavior to the evaporation of remaining alcohol and absorbed moisture and/or due to the successive expansion/shrinkage cycles during SLS process. However, this difference in morphology of the powder was not observed by Lopes et al. [25], but these authors only studied five printing cycles.

### 3.2. Printed Parts

**Figure 8:**
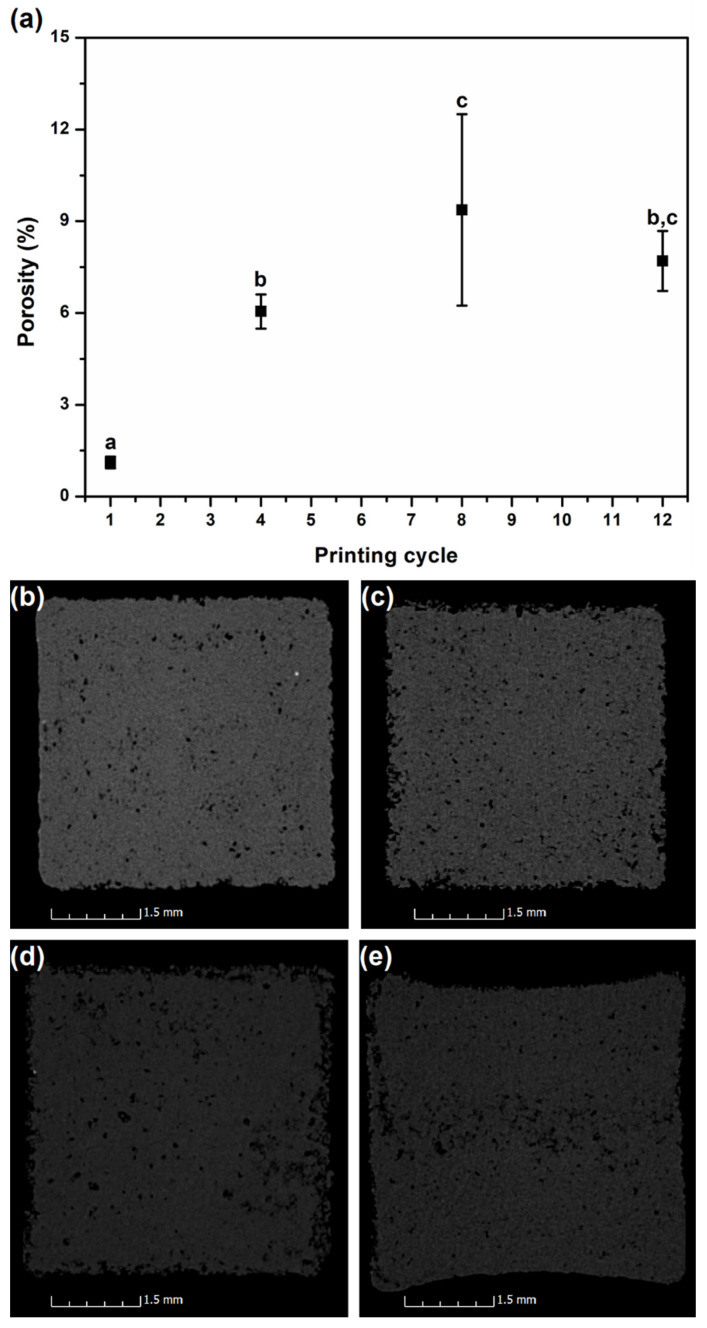
CT results: (**a**) porosity level for different printing cycles, where statistically homogenous groups are indicated by the same letters (a, b, and c); transversal cuts done through CT at 10 mm from the base of samples for different printing cycles: (**b**) 1; (**c**) 4; (**d**) 8 and (**e**) 12.

The porosity evolution of printed parts along the printing cycles, together with the transversal cuts made in each sample, is shown in Figure 8. Through the analysis of the 12 zones obtained from the 3 different cross-section cuts made in each sample, it is noticeable that the average porosity level of the samples increases with the number of reprocessing cycles, with statistically significant differences between almost all the reprocessing cycles. This is particularly evident in reprocessing cycle 8. This result might be related to the increased porosity observed in the powders from the first to the last cycle (see Figure 7). On the other hand, a decrease in porosity level from printing cycles 8 to 12 seems to occur. However, and as shown in Figure 8e, this is just apparent, since there is a clear deformation (depression) in the samples of printing cycle 12. This deformation might result from the collapse of the sample walls due to extreme local porosity. If this is the case, porosity is, therefore, only artificially diminished.

**Figure 9:**
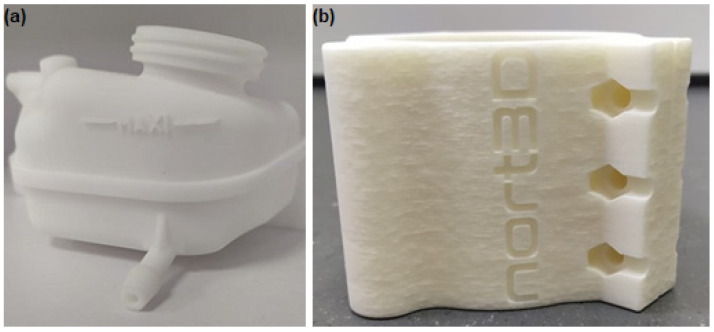
Commercial parts printed in parallel with the test samples used in this study for (**a**) printing cycle 1 and (**b**) printing cycle 12.

As the number of printing cycles increased, visual changes were also observed in the printed parts. Here, this is illustrated through the commercial parts that were produced in parallel with the studied test samples, where this effect was more evident. In Figure 9, a part printed in the beginning of the study (with 100% virgin powder) and another printed with 100% reused powder with 11 reprocessing cycles, are illustrated. As it can be seen, the printed part with virgin powder presents a white and smooth surface, while the printed part after 12 printing cycles shows a yellowish color and a much rougher surface. This phenomenon started to be evident after printing cycle 6.

Thus, considering the porosity level, color, and surface finishing of the printed parts, it was decided to limit the evaluation of the mechanical properties until printing cycle 6.

**Figure 10:**
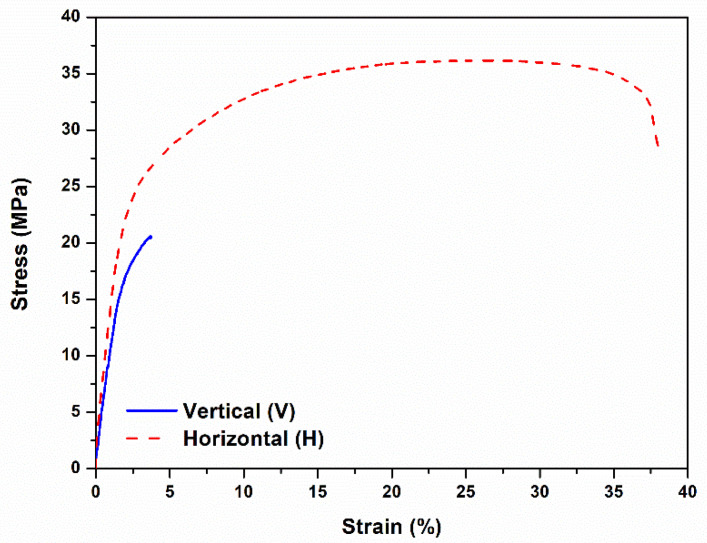
Representative stress–strain curves corresponding to the first printing cycle of tensile samples printed in horizontal (H) and vertical (V) orientations.

**Figure 11:**
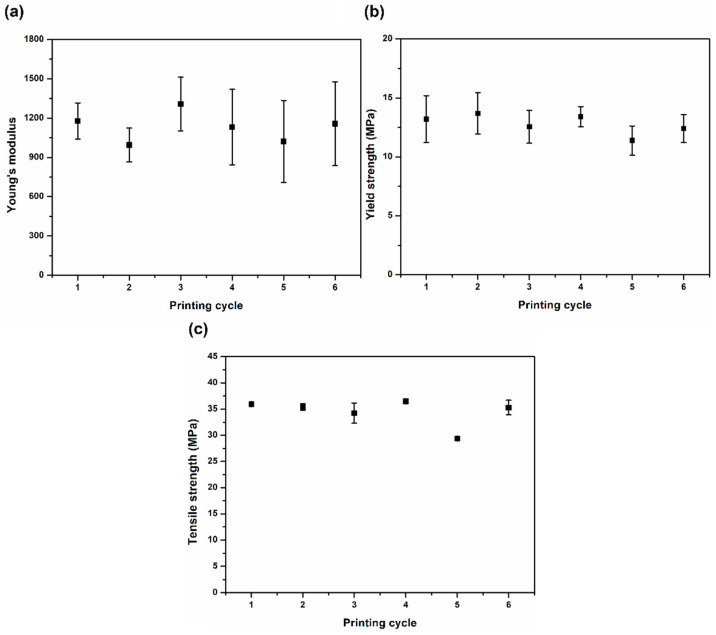
Tensile properties of samples printed in horizontal (H) orientation: (**a**) Young’s modulus, (**b**) yield strength and (**c**) tensile strength.

As referred before, the tensile samples were printed in two different orientations, horizontal (H) and vertical (V). Concerning the horizontal orientation, samples from all the cycles were analyzed. However, for the vertical orientation only some of the cycles were considered, once these samples had much lower mechanical performance (namely, yield and tensile strength) than those produced in the horizontal orientation, as can be seen in Figure 10. The relevant mechanical properties of all the samples are shown in Figure 11 and Table 4. As described in the literature, vertically printed samples presented a brittle behavior, barely reaching the plastic domain. This behavior is probably due to weak interlayer bonding, a drawback of the additive (layer by layer) manufacturing techniques [25,29]. In the V samples, these weak planes are perpendicular to the force applied in the tensile tests, promoting their early rupture.

Figure 11 shows the average values obtained for the different properties of the samples printed in the horizontal orientation, where no statistically significant differences were observed.

Concerning the H samples, it can be seen that the variations in the average values of the Young’s modulus and tensile strength along the printing cycles are not statistically significant. However, an increasing lack of homogeneity of the printed samples is apparent since the standard deviation values, especially in the case of the Young’s modulus, tend to increase with the printing cycle number. This can be an indication of powder inhomogeneity (that is already present in the first cycle), since the printing conditions remain the same. In fact, and as already referred, the quality of the power is expected to deteriorate more in the vicinity of the printed parts contour, where the laser promotes the required increase in temperature needed for sintering. Therefore, despite the powder post-processing treatment, some small agglomerates (with dimensions lower than 200 μm, in this case), resulting from environmental humidity absorption or from partial sintering, exist, inducing some inhomogeneity. Additionally, the thermal degradation of the powder particles can also be different due to differences in their thermal history and in their molecular weight.

Thus, the powder post-processing treatment after each printing cycle seems to be an effective way to minimize the negative impact of reusing the powder, valid until the sixth cycle. This constitutes an alternative to the addition of 20% or 30% of virgin powder after each printing cycle, as recommended by the powder supplier or by Lopes at al. [25], respectively. It is worth mentioning that those authors, who did not treat the powder, obtained a 20% decay in the mechanical properties after only 2 cycles, when using 100% of reused powder.

It is worth mentioning that the Young’s moduli obtained in the present work were always lower than that claimed by the powder supplier, as in [26].

## 4. Conclusions

SLS technology is a very versatile production process and enables to employ reused powder. However, a loss in properties reproducibility when 100% of reused PA 12 powder is used was observed. This inconsistency is not only a consequence of the different thermal histories of the (re)used powder particles, but also inherent to the process, as can be seen from the properties scatter of the samples printed in the first cycle.

The degradation of the powder with successive use is apparent. The DSC analysis showed a clear decrease in the melting enthalpy and a slight decrease in the melting temperature, meaning that the degree of crystallization is gradually decreasing throughout the printing cycles. This might indicate the occurrence of chain scission, since shorter chains have higher mobility, which makes the crystallization process more difficult.

Moreover, the degradation of the PA 12 powder was observed by SEM: after 12 printing cycles, the surface of the powder is rougher and seems to have an increased porosity when compared with the virgin powder.

CT analysis results are in line with the powder morphology obtained by SEM, i.e., an increase in printed parts porosity is observed along the printing cycles. This phenomenon is extremely negative for functional parts since it may be responsible for their premature failure and/or for their lack of dimensional accuracy. These negative features are mainly evident after the 6th reprocessing cycle. The visualization of printed parts with virgin and reused powder shows that smooth and clear surfaces are nearly impossible to obtain with powder subjected to more than 6 printing cycles.

The mechanical characterization showed that the mechanical properties loss is not significant until the 6th printing cycle, but that the corresponding standard deviation increases. Moreover, this characterization showed that the parts printed vertically have worse mechanical performance than those printed horizontally. Therefore, vertical printing orientation should be avoided whenever mechanical functionality is required.

Lastly, probably the most significant conclusion of this work is that the post-processing treatment of the (re)used powder, namely, through milling, filtering, and homogenizing, minimizes its degradation by eliminating the most problematic powder (agglomerates) and homogenizing the remaining powder. This powder post-processing treatment constitutes, therefore, an alternative to the approach used by Lopes et al. [25] that consisted in the addition of virgin powder after each printing cycle.

## Figures and Tables

**Table 1:** PA12-L 1600 powder properties.

Property	Value
Bulk density (g/cm^3^)	0.48
Density of solid parts (g/cm^3^)	0.95
Melting temperature (°C)	183
Tensile strength (MPa)	46
Young’s modulus (MPa)	1602
Elongation at break (%)	36

**Table 2:** General characteristics of the ProMaker P1000 equipment.

Specification	Value
Max. build volume (mm^3^)	300 × 300 × 300
Laser power (CO_2_) (W)	30
Max. temperature (°C)	200
Precision (mm)	0.02

**Table 3:** SLS processing parameters.

Parameter	Value
Feed cartridges temperature (°C)	135
Pistons temperature (°C)	135
Build platform temperature (°C)	180
Purge Nitrogen temperature (°C)	120
Layer thickness (mm)	0.1
Layering roller speed (mm/s)	140
Laser Speed (mm/s)	3.5
Infill laser parameters	
Laser power (W)	14
Laser beam offset (mm)	0.75
Hatching distance (mm)	0.2
Outline laser parameters	
Laser power (W)	10
Laser beam offset (mm)	0.54

**Table 4:** Tensile properties of samples printed in horizontal (H) and vertical (V) orientations for different printing cycles.

Printing Cycle and Printing Orientation	Young’s Modulus (MPa)	Yield Strength (MPa)	Tensile Strength (MPa)
1-H	1178.14 ± 138.31	13.20 ± 2.00	35.95 ± 0.43
2-H	994.98 ± 128.51	13.69 ± 1.77	35.42 ± 0.60
3-H	1306.87 ± 205.39	12.56 ± 1.40	34.23 ± 1.95
4-H	1131.78 ± 287.59	13.41 ± 0.85	36.48 ± 0.22
5-H	1021.98 ± 312.92	11.39 ± 1.23	29.35 ± 0.40
6-H	1156.76 ± 318.55	12.40 ± 1.20	35.28 ± 1.39
1-V	894.22 ± 108.84	5.78 ± 1.73	21.86 ± 1.24
3-V	1101.00 ± 270.6	4.01 ± 1.52	24.45 ± 0.81
6-V	1175.97 ± 683.09	2.83 ± 0.90	17.90 ± 0.74
